# Fatigue Resistance of Customized Implant-Supported Restorations

**DOI:** 10.3390/ma18143420

**Published:** 2025-07-21

**Authors:** Ulysses Lenz, Renan Brandenburg dos Santos, Megha Satpathy, Jason A. Griggs, Alvaro Della Bona

**Affiliations:** 1 Postgraduate Program in Dentistry, School of Dentistry, University of Passo Fundo, Passo Fundo 99052-900, RS, Brazil; 159584@upf.br (U.L.);; 2 Department of Biomedical Materials Science, School of Dentistry, University of Mississippi Medical Center, Jackson, MS 39216, USA

**Keywords:** dental implants, custom abutments, finite element analysis, fatigue behavior, endurance limit

## Abstract

The design of custom abutments (CA) can affect the mechanical reliability of implant-supported restorations. The purpose of the study was to evaluate the influence of design parameters on the fatigue limit of CA and to compare optimized custom designs with the reference abutment (RA). A morse-tapered dental implant, an anatomical abutment, and a connector screw were digitalized using microcomputed tomography. A cone beam computed tomography scan was obtained from one of the authors to virtually place the implant-abutment assembly in the upper central incisor. Ten design parameters were selected according to the structural geometry of the RA and the implant planning. A reverse-engineered RA model was created in SOLIDWORKS and was modified considering a Taguchi orthogonal array to generate 36 CAs with ±20% dimensional variations. Finite element analysis was conducted in ABAQUS, and fatigue limits were estimated using Fe-safe. ANOVA (α = 0.1) identified the most influential parameters. Von Mises stress values ranged from 229 MPa to 302 MPa, and 94.4% of the CAs had a higher fatigue limit than the RA. Three parameters significantly affected the fatigue performance of the implant system. The design process of custom abutments includes critical design parameters that can be optimized for longer lifetimes of implant-abutment restorations.

## 1. Introduction

Tooth replacement requires a complex treatment planning process, and implant-retained restorations are the gold standard for achieving satisfactory functional and biological outcomes [1]. Immediate implant placement has demonstrated promising hard tissue responses, favorable Pink Esthetic Scores, and high success rates [2]. High primary stability and immediate loading of implant-retained restorations have been reported as key factors in maintaining peri-implant tissue health, meeting modern society’s rising esthetic standards, and providing faster treatments [3]. However, the challenges in promoting long-term peri-implant soft tissue stability highlight the importance of the transcrestal design of the restoration and abutment. Optimal prosthetic management should consider the design profile at different peri-implant levels, which varies according to the 3D implant position, alveolar anatomy, and tooth region [4,5,6]. Achieving the ideal emergence profile design requires personalized planning for each patient [3,7].

Using custom rather than prefabricated abutments is a promising alternative for optimizing the treatment process [8,9]. This procedure enables the prosthodontist to adapt the abutment design based on each patient’s specific anatomy, resulting in peri-implant soft tissue stability and long-term success [10]. Clinical studies have used the anatomy of the tooth to be extracted or the contralateral tooth (visualized through CBCT images and implant-guided surgery software CoDiagnostix 2024 10.8) as a reference for designing the custom abutment. Among the evaluated parameters, an increase in bucco-palatal width and transmucosal height was the most frequently observed modification [11,12]. Personalized abutments can also be manufactured from a variety of materials, allowing for functional restorations and convenience with chairside digital workflows [13]. Despite these advantages, further standardization of the manufacturing process is required to enhance the mechanical reliability, as some studies demonstrated higher fracture strength for prefabricated over custom abutments [14,15].

A variety of prosthetic abutments are currently available, and each brand offers distinct designs for abutment preparations, transmucosal regions, prosthetic finishing lines, and implant-abutment connections [16,17]. Several studies have evaluated the mechanical behavior of different implant-abutment connections [18,19,20] and abutment transmucosal heights [17,21] using finite element analysis (FEA). These parameters are often adjusted according to different clinical scenarios, with the recommendation of optimizing to the best geometry, which remains challenging to accomplish. Understanding the fatigue behavior of implant-abutment systems is crucial for establishing the longevity of oral rehabilitation [22,23], and the fatigue limit (an outcome that can be predicted using virtual simulations) defines the load at which the abutment would have an infinite lifetime [24]. In terms of optimization processes, studies primarily focused on improving the mechanical behavior of implant bodies using the response surface method and design of experiments (DOE) in virtual simulations, yielding favorable results [25,26]. Conversely, for abutments, current design optimization outcomes are restricted to bone remodeling for a custom zirconia infrastructure cemented to a titanium base abutment [27]. Although a recent study assessed the effect of various dental implant design parameters [28], no study investigated the impact of customizing the abutment design.

These factors are fundamental during the treatment planning process, yet there is insufficient information regarding the effects of different design parameters on the fatigue behavior and load distribution of abutments. Because customized abutments consider the patient’s anatomy, these parameters could potentially be optimized to provide the best abutment design for each clinical scenario. Therefore, the objective of this study is to evaluate the influence of design parameters on the fatigue limit of custom abutments and to compare optimized custom designs with the reference abutment using FEA. The hypotheses are that variations in abutment width and height influence the fatigue limit of the implant system, and the design can be optimized to outperform a prefabricated abutment.

## 2. Method

### 2.1. Digitalization of Structures

A Morse-tapered dental implant (Helyx, Grand Morse, Neodent, 3.5 mm in diameter and 16 mm in height), a prefabricated abutment (anatomical abutment, narrow body with 2.5 mm of transmucosal height, Neodent), and a connector screw were scanned using microcomputed tomography (Micro-CT) (Skyscan 1172; Micro Photonics, Allentown, PA, USA) with both aluminum and copper filters and a pixel size of 34.6 μm. The generated 3D bitmap files were processed with a grayscale thresholding tool to create digital replicas of the physical specimens using Simpleware W-2024.12 (Synopsis, Sunnyvale, CA, USA). The resulting 3D models were used to measure the geometry of the components.

**Figure 1:**
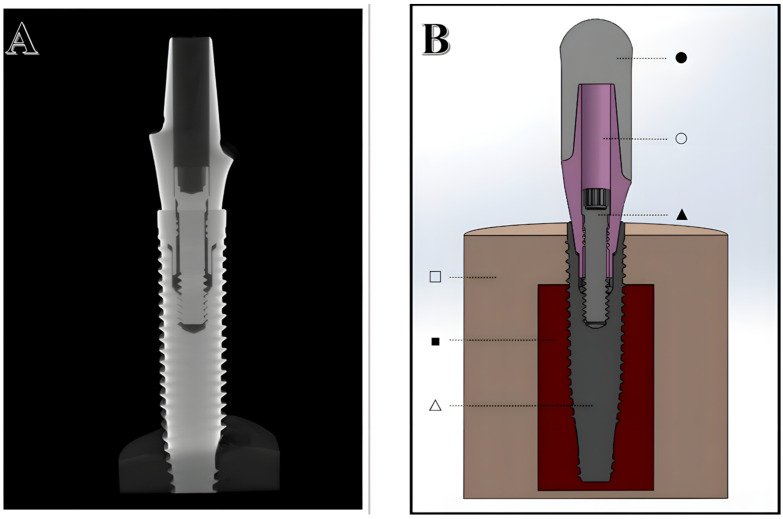
(**A**) Buco-palatal cross-section of the implant-abutment assembly obtained from a micro-CT scan. (**B**) Buco-palatal cross-section of the SOLIDWORKS assembly created from digitally modeling the physical specimens. The components in (**B**) are as follows: loading cap (● pearl white), reference abutment (○ pink), connector screw (▲ light gray), implant body (△ dark gray), cortical bone (□ beige), and cancellous bone (■ dark red).

Computer-aided design (CAD) software (SOLIDWORKS 2023, D’assault Systèmes, Vélizy-Villacoublay, France) was used to digitally replicate the physical specimens with the previously measured dimensions, applying a reverse-engineering technique. The implant’s tapered internal connection and the hexagon indexation from the reference abutment (RA) were maintained for all generated abutments. A hemispherical loading cap and a simulated specimen holder with cortical and cancellous layers were constructed [22,23,29,30]. The SOLIDWORKS parts were assembled to match the physical assembly, and the implant was positioned 0.8 mm above the cortical bone level to simulate the worst-case scenario for an internally tapered connection. This experimental characteristic was adopted considering that the bone loss observed in a cohort with 10,871 implants with 8–10 years of follow-up was 0.49 ± 0.74 mm [1]. Yet, this type of implant connection is usually recommended to be placed below bone level. The resulting SOLIDWORKS assembly and a 2D image obtained from the micro-CT scan of the physical assembly are shown in Figure 1.

Software for guided surgery planning (CoDiagnostiX 2024 10.8, Straumann, Oslo, Norway) was used to place the implant-abutment assembly in the upper right central incisor based on a cone beam computerized tomography (CBCT) scan from one of the authors (U.L.). The virtual 3D positioning of the implant followed 4 important factors [7]: implant depth (4 mm below the gingival margin); interproximal position (centered with the mesio-distal distance of the tooth); implant body position (slightly palatalized); and axial inclination (screw access positioned between the cingulum and the incisal edge). After positioning the implant-abutment assembly, the patient-related parameters were measured using the distance measurement tool from CoDiagnostiX software.

### 2.2. Design of Experiments

**Figure 2:**
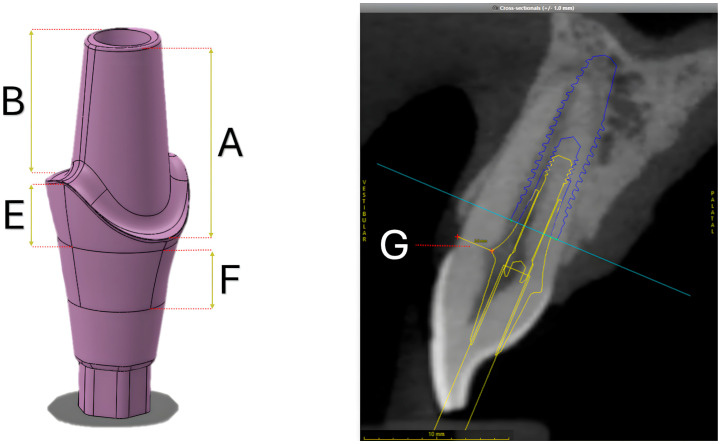
Schematic representation of the design parameters presented in Table 1. (**Left**): Reference abutment model generated from SolidWorks illustrating design parameters A, B, E, and F. (**Right**): Sagittal cross-section from the CoDiagnostiX plan demonstrating the relation with the abutment’s finishing line and the teeth’s buccal margin (parameter G).

Ten design parameters were screened and selected considering the reference abutments’ geometry (6 parameters) and the patient-related data (4 parameters) (Figure 2). The design parameters were selected based on concepts of biologic space within the implant–abutment system and based on commonly observed prosthetic design variations among available prefabricated abutments. The design parameters were independent of each other, allowing for the evaluation of the most influential factors affecting the abutment geometry. The design parameter values were measured from physical and digital replicas using an optical microscope and SOLIDWORKS, respectively, and the patient-related parameter values were obtained from measurements in the guided surgery plan. These values were defined as reference values, from which two additional levels were created by increasing or decreasing the original value by 20% because this provided three levels of each design factor to investigate non-linear effects. For the patient-related parameters, only the reference and lower values were considered. The objective of the customization process is to ensure that the abutment occupies more 3D space within the alveolus, and a higher distance from the dentin margin to the abutment’s finishing line would result in a narrower abutment. Therefore, the higher values for the four patient-related design parameters were excluded from this experiment. Patient-related parameters systematically changed the width of the abutment at the transmucosal level (with a concave profile), as well as at the abutment preparation and prosthetic finishing platform levels, resulting in a wider abutment. The ten design parameters and their corresponding values can be seen in Table 1.

To create different designs of custom abutments, a Design of Experiments (DOE) was performed in Reliasoft (DOE++) using a Taguchi Orthogonal Array with three levels (reference, 20% lower, and 20% higher) for parameters A to F and two levels (reference and 20% lower) for parameters G to J. The experiment resulted in 36 combinations of geometrical values based on the ten design parameters and their respective levels (Figure 2). Therefore, 36 abutment designs were modeled in SOLIDWORKS and assembled with the reference components (simulated cortical and cancellous bone, implant body, connector screw, and loading cap) to test the fatigue limit of each system. ANOVA was used to screen the design parameters that had significant effects on the implant fatigue limit (α = 0.1). Lenth’s method was used to estimate the variance of the effects. Under this methodology, the estimated variance is calculated as 1.5 times the median value of all effects that are less than 2.5 s_0_, where s_0_ is 1.5 times the median of all effects [31].

### 2.3. Virtual Mechanical Testing

Finite element analysis (FEA) was performed in ABAQUS 2023 (SIMULIA, D’assault Systèmes) for the implant mechanical test having the geometry described in the ISO 14801 standard. The SOLIDWORKS models were exported to ABAQUS, where the material properties were defined as isotropic and homogeneous. Young’s modulus and Poisson’s ratio were assigned based on data from Table 2. A surface-to-surface interaction was applied to the transfixing screw and implant contact surfaces. A strong interface resulting from the application of preload torque to the connector screw was considered in the simulation of the implant–abutment interaction at the Morse taper internal connection [22,32]. Osseointegration was simulated using a tie constraint between the implant threads and the cortical and cancellous bone. Boundary conditions were applied to the outer surface of the cortical bone to prevent movements after load applications, as physiologically occurs. An initial load of 100 N was applied to the palatal surface of the loading cap at an angle of 30 degrees relative to the implant axis. The models were meshed using tetrahedral linear elements. A convergence study was conducted on the reference model using different element sizes. The mesh size that provided the shortest computational time without compromising accuracy was 0.15 μm. The reference model had 614,678 elements.

The .odb files generated from the FEA tests were imported into the fe-safe 2023 post-processing software (SIMULIA, D’assault Systèmes). The stress increments calculated in ABAQUS were used to simulate cyclic loading according to the ISO 14801 standard [37], with a frequency of 2 Hz and a stress ratio of 0.1. The Brown-Miller criterion, with Morrow’s mean stress correction, was applied to estimate the model’s fatigue life [38]. To determine the fatigue limit, an initial load of 100 N was applied, resulting in an infinite fatigue life. Subsequently, a load of 200 N was applied, which produced a finite fatigue life. Intermediate loads were then tested to identify the threshold load value at which fatigue life transitioned from finite to infinite. This threshold represents the maximum load at which the specific abutment design would have an infinite fatigue life considering the experimental conditions. On average, five intermediate loads were tested to determine the fatigue limit of each abutment design.

## 3. Results

**Figure 3:**
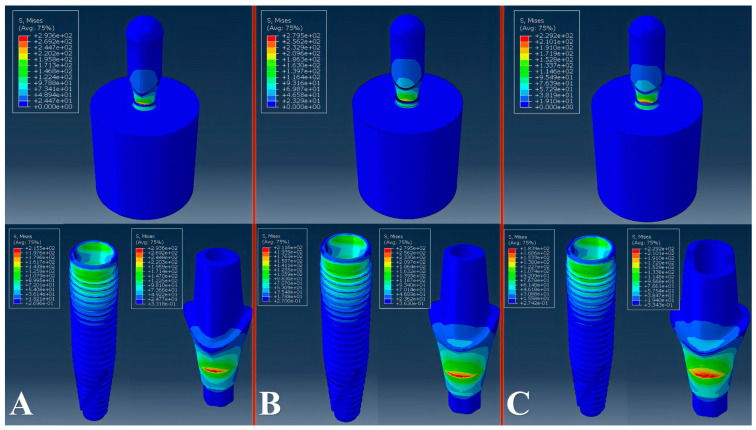
Von Mises stress distribution (in MPa) for the entire model (top) and separately for the implant and abutment (bottom). (**A**): Custom abutment with the lowest fatigue limit (CA 06); (**B**): Reference abutment; (**C**): Custom abutment with the highest fatigue limit (CA 34). All models were subjected to a 100 N load.

The peak von Mises stress of the models subjected to 100 N of load was concentrated above the implant platform, at the buccal region of the reference and custom abutments (Figure 3). The peak von Mises stress values ranged from 229 MPa to 302 MPa. The abutment, implant, and connector screw, in this order, received the highest von Mises stresses within the evaluated models. All evaluated models showed an infinite lifetime with 100 N of load. The custom abutment #34 showed the highest predicted fatigue limit: 173.5 N, while the reference abutment showed 140 N. The fatigue limits for the evaluated abutments can be seen in Table 3. The majority (94.4%) of the custom abutments showed higher fatigue limits than the reference abutment.

**Figure 4:**
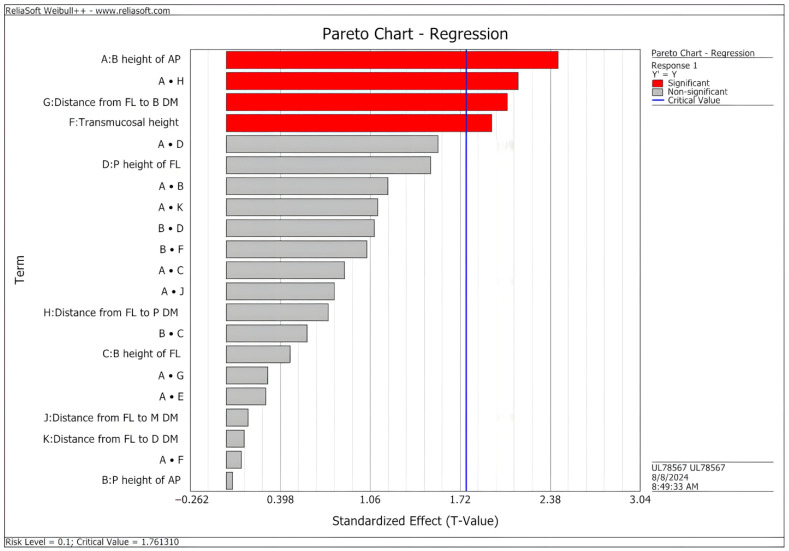
Pareto regression chart with the significant design parameters (standardized effect > 1.76) on the fatigue limit of custom abutments. When the characters “•” appear between parameters, they indicate two-way interactions. Abbreviations: B: buccal; P: palatal; M: mesial; D: distal; AP: abutment preparation; FL: finishing line; DM: dentin margin.

The optimal fatigue limit was predicted when parameters A (buccal height of the abutment preparation), F (transmucosal height), and G (distance from the prosthetic finishing line to buccal dentin margin) had the lowest values, and these three parameters showed a significant effect (standardized effect > 1.76). The two-way interaction of parameters A and H (distance from the prosthetic finishing line to the palatal dentin margin) was also significant (Figure 4).

## 4. Discussion

Designing the geometry of dental abutments to achieve optimal mechanical and biological outcomes is a complex task for both manufacturers and dentists. For custom abutments, the challenge increases as the design considers patient-specific anatomy. This study investigated critical design parameters to reduce the variability of custom abutments, aiming to benefit the dental community, including dental companies, technicians, dentists, and patients. Each design parameter chosen for this computational experiment has direct clinical relevance in guiding the selection of a proper abutment. The present study showed that the design of abutments can be optimized to improve the fatigue behavior of the implant complex, with three critical design parameters. Thus, the hypothesis of the study was accepted.

The buccal height of the abutment preparation (parameter A) was a significant design factor, showing a higher fatigue limit with shorter heights. Such a relationship is in agreement with a previous finite element analysis study [39], which demonstrated that longer abutment preparations induced greater deformations in the implant-abutment system. Consistently, Hendi et al. [40] observed that longer abutment preparations experienced increased screw loosening when subjected to cyclic loading. Another critical parameter was the transmucosal height (parameter F), which demonstrated higher fatigue limits at lower heights. This behavior may be attributed to a shorter transmucosal emergence profile in the buccal region of the abutment geometry, where peak von Mises stresses occurred. Increasing the titanium material volume in such a region could improve fatigue resistance. Similar results were reported in previous in-vitro and FEA studies [17,21,41]. In addition, some studies also showed that the transmucosal height influenced marginal bone loss and peri-implantitis [42,43]. Notably, we ensured that the moment arm for all abutments was standardized by adjusting the height of the loading cap, as instructed by ISO 14801.

A patient-related design parameter (G) was significant for the fatigue limit in the buccal region, probably because of an increased amount of titanium in an area with stress concentration. As lingual implant placement is recommended, the distance between the finishing line and the buccal dentin margin increases, leading to a greater addition of titanium material when parameter G had the lowest values. Furthermore, the interaction between parameters A and H (palatal distance) also had a significant effect on the fatigue limit. A more extended palatal profile, combined with reduced buccal height, may create a geometry that minimizes stress concentration in critical regions. However, these interpretations should be considered with caution, as this study did not include detailed load distribution analysis. In addition, the reasons for parameters I and J (mesial and distal distances, respectively) not being significant could be related to insufficient space between the finishing line and the corresponding dentin margin, limiting the possibility of a wider abutment. Therefore, in regions with larger alveolus dimensions, such as molars, these parameters may have higher relevance to the fatigue limit of custom abutments and should be further investigated. It is important to note that parameters A-J were measured based on the geometry of a prefabricated abutment. In clinical practice, however, abutment design should account for the occlusal relationship, transmucosal level, and soft tissue positioning. Consequently, because these design parameters should be patient-specific, we suggest that the fabrication of custom abutments should follow a well-established design optimization process combined with guided implant planning to advance the state of the art of implant-supported restorations. Although the angulation of the screw access channel may be required for specific treatments, it represents a risk to the biomechanical properties of the implant-abutment system [44]. This design parameter was not considered in this study because it would require different implant 3D placements and restrict most other design factors.

Recent in-vitro studies comparing the mechanical properties between customized and prefabricated abutments showed conflicting results [14,15,45,46,47]. The present study suggests two major reasons for these discrepancies. First, the unpredictability in the manufacturing process of custom abutments may lead to variability in mechanical durability. The design factors affecting the mechanical properties of abutments, as observed in the present study, can have either a positive or negative effect on fatigue resistance, depending on the specific design used in each study. Second, compliance with the ISO standard [37] can influence the outcome, so the present study simulated a clinically relevant 0.8 mm of bone resorption [1] rather than the 3 mm worst-case scenario that is often used in in-vitro testing. Previous studies using FEA showed that the amount of marginal bone loss around dental implants influences the load distribution of the system [48,49]. Therefore, further in-vitro studies should investigate the fatigue performance comparing custom and prefabricated abutments, considering the most critical parameters in designing abutments for implant-supported restorations.

Virtual simulations can be valuable when designed to address recurring clinical problems. Although these technologies are still in their early phases of direct clinical evidence, they can assist us in increasing the mechanical behavior of high-cost dental materials. This, in turn, can guide decision-making in robust clinical studies, assist companies in optimizing their manufacturing processes, and help dentists in designing custom abutments more effectively. However, there are a few study limitations, such as the evaluation of a single clinical case for a central incisor. Both the alveolus anatomy of central incisors and the 3D implant position can vary in each clinical scenario. Therefore, caution is advised when extrapolating these results to other tooth regions. Further studies on different design parameters and alveolar anatomies are encouraged.

## 5. Conclusions

Considering the results of the study, it can be concluded that:

- The analysis of customized abutments identified design factors that have significant effects on the fatigue behavior of implant-abutment systems, as predicted by virtual modeling;
- Lower abutment preparation and transmucosal heights increased the predicted fatigue limit of the implant-abutment system;

Individualized CBCT-based planning can guide abutment geometry optimization to enhance mechanical longevity, within the limits of each clinical case.

## Figures and Tables

**Table 1:** Design parameters and their respective measurements (in mm).

Design Parameters		20% Lower	Reference	20% Higher
**A**	Buccal height of the abutment preparation	4.25	5.32	6.38
**B**	Palatal height of the abutment preparation	3.41	4.26	5.11
**C**	Buccal height of the finishing line	0.16	0.2	0.24
**D**	Palatal height of the finishing line	1.28	1.6	1.92
**E**	Interproximal height of finishing line	1.64	2.05	2.46
**F**	Transmucosal height	1.6	2	2.4
**G**	Distance from the prosthetic finishing line to buccal dentin margin	2.06	2.57	N/A
**H**	Distance from the prosthetic finishing line to palatal dentin margin	0.6	0.77	N/A
**I**	Distance from the prosthetic finishing line to mesial dentin margin	0.54	0.67	N/A
**J**	Distance from the prosthetic finishing line to distal dentin margin	0.5	0.62	N/A

N/A: non-applicable.

**Table 2:** Material properties assigned to each component used in the finite element models.

Component	Material	Young’s Modulus (GPa)	Poisson’s Ratio	Ultimate Tensile Strength (MPa)
Cap	Zirconia [33]	205	0.30	-
Abutment	Ti 6Al-4V ELI [34]	113.8	0.31	825
Connector screw	Ti 6Al-4V ELI [34]	113.8	0.31	825
Implant body	CP Ti Grade 4 [35]	103	0.34	660
Cortical bone	Cortical bone [36]	20	0.30	-
Cancellous bone	Cancellous bone [36]	14	0.30	-

Ti: Titanium; Al: Aluminum; V: Vanadium; ELI: extra low interstitial; CP: commercially pure.

**Table 3:** Maximum von Mises stress (in MPa) using 100 N of load and the predicted fatigue limits (in N) for the evaluated abutments with their respective design parameters (in mm).

Evaluated Abutment	Parameter A	Parameter B	Parameter C	Parameter D	Parameter E	Parameter F	Parameter G	Parameter H	Parameter I	Parameter J	Peak Von Mises Stress	Fatigue Limit
RA	6.25	6.25	0.2	1.6	2.05	2	2.57	0.77	0.67	0.62	279	140
CA 1	5	5	0.2	1.6	1.64	2	2.06	0.6	0.67	0.62	260	165
CA 2	6.25	5	0.16	1.6	2.05	1.6	2.57	0.6	0.54	0.62	242	137
CA 3	5	6.25	0.2	1.28	1.64	2	2.06	0.6	0.54	0.5	302	142
CA 4	7.5	6.25	0.2	1.28	2.46	2	2.06	0.6	0.54	0.5	288	149
CA 5	7.5	5	0.16	1.6	2.46	1.6	2.57	0.6	0.54	0.62	252	144
CA 6	6.25	6.25	0.2	1.28	2.05	2	2.06	0.6	0.54	0.5	293	133
CA 7	7.5	5	0.16	1.28	2.46	1.6	2.06	0.6	0.54	0.5	242	163
CA 8	7.5	5	0.2	1.6	2.46	2	2.06	0.6	0.67	0.62	282	146
CA 9	5	6.25	0.16	1.6	1.64	2	2.06	0.77	0.54	0.62	252	168
CA 10	5	6.25	0.16	1.28	1.64	1.6	2.06	0.77	0.67	0.62	254	170
CA 11	5	6.25	0.16	1.6	1.64	2	2.57	0.6	0.67	0.5	263	147
CA 12	7.5	6.25	0.16	1.28	2.46	1.6	2.06	0.77	0.67	0.62	283	147.5
CA 13	7.5	6.25	0.16	1.6	2.46	2	2.57	0.6	0.67	0.5	263	145
CA 14	7.5	6.25	0.2	1.6	2.46	1.6	2.57	0.77	0.54	0.5	255	142
CA 15	6.25	5	0.16	1.28	2.05	2	2.57	0.77	0.67	0.5	275	147
CA 16	7.5	6.25	0.16	1.6	2.46	2	2.06	0.77	0.54	0.62	288	155
CA 17	5	5	0.2	1.28	1.64	2	2.57	0.77	0.54	0.62	254	156
CA 18	5	5	0.2	1.6	1.64	1.6	2.06	0.77	0.67	0.5	263	152
CA 19	7.5	5	0.2	1.6	2.46	1.6	2.06	0.77	0.67	0.5	273	146
CA 20	6.25	5	0.16	1.28	2.05	1.6	2.06	0.6	0.54	0.5	271	149
CA 21	6.25	5	0.2	1.28	2.05	2	2.57	0.77	0.54	0.62	284	141
CA 22	6.25	5	0.2	1.6	2.05	2	2.06	0.6	0.67	0.62	280	141
CA 23	5	5	0.16	1.28	1.64	1.6	2.06	0.6	0.54	0.5	262	143
CA 24	6.25	6.25	0.16	1.6	2.05	2	2.06	0.77	0.54	0.62	282	155
CA 25	6.25	5	0.2	1.6	2.05	1.6	2.06	0.77	0.67	0.5	247	164
CA 26	7.5	6.25	0.2	1.28	2.46	1.6	2.57	0.6	0.67	0.62	271	141.5
CA 27	7.5	5	0.2	1.28	2.46	2	2.57	0.77	0.54	0.62	244	150
CA 28	5	5	0.16	1.28	1.64	2	2.57	0.77	0.67	0.5	257	145.5
CA 29	5	6.25	0.2	1.28	1.64	1.6	2.57	0.6	0.67	0.62	258	143.5
CA 30	7.5	5	0.16	1.28	2.46	2	2.57	0.77	0.67	0.5	265	141
CA 31	6.25	6.25	0.2	1.6	2.05	1.6	2.57	0.77	0.54	0.5	238	163.5
CA 32	5	5	0.16	1.6	1.64	1.6	2.57	0.6	0.54	0.62	250	144
CA 33	6.25	6.25	0.16	1.28	2.05	1.6	2.06	0.77	0.67	0.62	264	167
CA 34	5	6.25	0.2	1.6	1.64	1.6	2.57	0.77	0.54	0.5	229	173.5
CA 35	6.25	6.25	0.16	1.6	2.05	2	2.57	0.6	0.67	0.5	260	148
CA 36	6.25	6.25	0.2	1.28	2.05	1.6	2.57	0.6	0.67	0.62	238	154

RA: Reference abutment; CA: Custom abutment.

## Data Availability

The original contributions presented in this study are included in the article. Further inquiries can be directed to the corresponding authors.
